# Perforated peptic duodenal ulcer in a paraesophageal hernia – a case report of a rare surgical emergency

**DOI:** 10.1186/1471-2482-6-1

**Published:** 2006-01-26

**Authors:** Mikael Ekelund, Else Ribbe, Julian Willner, Thomas Zilling

**Affiliations:** 1 Department of Surgery, Lund University Hospital, SE-221 85 Lund, Sweden; 2 Department of Imaging and Clinical Physiology, Lund University Hospital, SE-221 85 Lund, Sweden

## Abstract

**Background:**

Paraesophageal hernias are quite common and sometimes feared due to the risk of incarceration and strangulation of any herniated organ. The hereby reported combination of an incarcerated paraesophageal hernia containing a perforated peptic ulcer is extremely rare.

**Case presentation:**

An elderly man with multiple medical conditions was admitted due to severe upper abdominal pain. The patient was found to have a paraesophageal hernia and underwent a laparotomy. In the hernia, a perforated benign peptic duodenal ulcer was found. The duodenal defect was over-sewn, the hernial defect was closed and the former hernial cavity was drained by a right-sided chest tube. The patient was discharged one month after surgery and was found to do well at follow-up one month after discharge.

**Conclusion:**

This is the first report of a patient surviving the extremely rare and life-threatening combination of a perforated peptic duodenal ulcer in a paraesophageal hernia.

## Background

The distinction between a sliding hiatal hernia and a paraesophageal hernia is based on whether the esophagogastric junction (cardia) is above (sliding hernia or type I hiatal hernia) or below (paraesophageal hernia) the diaphragm. Paraesophageal hernias are true hernias with a covering peritoneal sac. Alternative names found are type II hiatal hernia, rolling hiatal hernia, intrathoracic stomach and up-side down stomach. A paraesophageal hernia with a sliding component is often termed a type III hernia.

Since paraesophageal hernias present in adult life in the majority of cases, acquired causes such as mechanical forces and tissue degeneration are probable etiological factors, albeit congenital factors cannot be ruled out since paraesophageal hernias and its complications also exist in the pediatric literature [1-5]. The incidence of paraesophageal hernias in per cent of all hiatal hernias ranges between 0, 5–19 % depending on whether or not to include mixed hernias, author's definition of paraesophageal hernia, and whether to include allcomers or only surgically treated patients [6].

The clinical presentation of paraesophageal hernias may be of wide range, from an incidental finding at one hand, to a catastrophic and life threatening condition at the other. If strangulation is suspected, decompression by a naso-gastric tube may buy time to surgery. Besides strangulation of the paraesophageal hernia, emergency surgery may also be required in patients presenting with hemorrhage due to a gastric ulcer or in patients with a perforation of the neck of the hernia.

A perforated peptic ulcer exposes the patient to a considerable risk of dying particularly in patients above seventy years of age. These patients require immediate surgery since there is a direct relationship between time from onset of symptoms to surgery vs. mortality and length of hospital stay [7].

We hereby report a case with the extremely rare combination of perforated peptic duodenal ulcer in a paraesophageal herniation with, for the first time in the literature, successful outcome.

## Case presentation

This 88-year-old man had several serious medical conditions: angina pectoris, chronic obstructive pulmonary disorder, type II diabetes, infected venous leg ulcers and a previous history of transurethral resection of the prostate, percutaneous coronary artery intervention and bilateral hip replacements. After a hip replacement 9 years ago the patient suffered from pulmonary embolization. Furthermore, there was a history of a conservatively treated bleeding gastric ulcer five years earlier.

**Figure 1:**
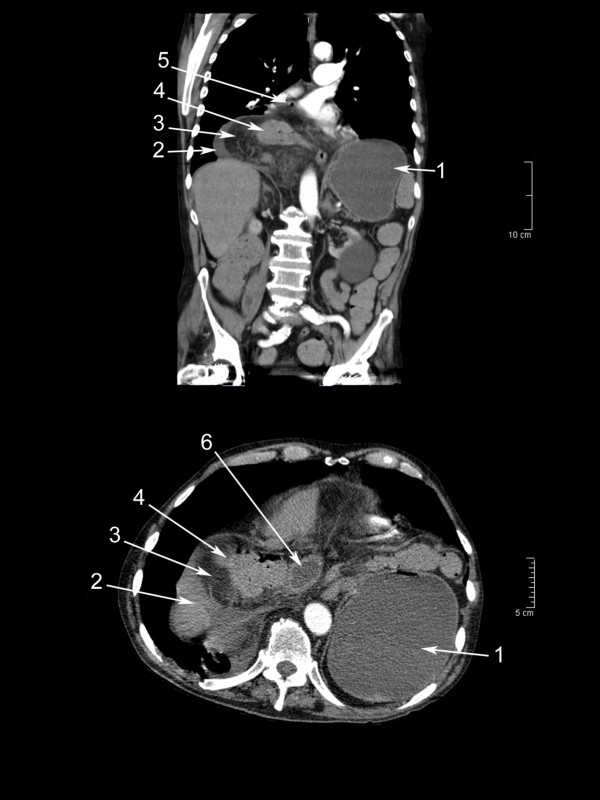
**Computed tomography on the day of arrival**. Frontal and transverse projections showing: 1. Intraabdominally distended gastric fundus. 2. Fluid in hernia. 3. Herniated major omentum. 4. Herniated transverse colon. 5. Small bubble of free air, not noticed primarily. 6. Herniated part of duodenum.

Shortly after having had supper the patient was suddenly struck by severe epigastric pain and an intense nausea with a few vomits and frequent belching. The patient arrived at the emergency department in pre-chock. His abdomen was soft and non-distended but with severe tenderness to palpation in the epigastrium. A CT scan of thorax and abdomen showed a paraesophageal herniation into the right thorax containing a part of the transverse colon and a part of the distal stomach and proximal duodenum. A pronounced gastric distension was noted. No free fluid or gas was found in the abdominal cavity. Besides the herniation and an abundance of faecal content in the entire colon, the CT scan was normal (Fig. 1).

**Figure 2:**
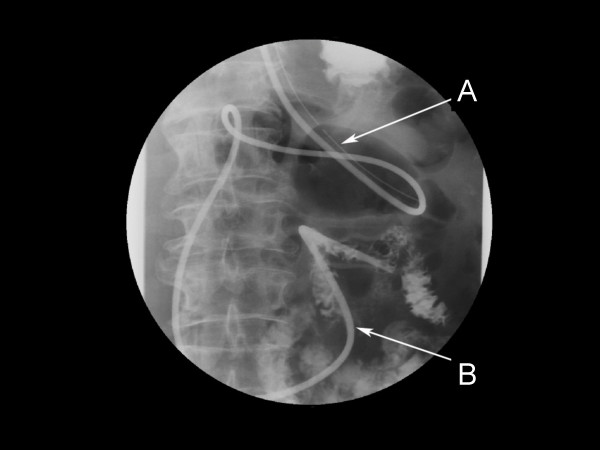
**Plain X-ray showing placements of tubes**. Naso-gastric tube in the intraabdominally located gastric fundus (A). The naso-jejunal tube was passed through the intrathoracic hernia and the end was placed in the proximal jejunum (B).

The patient received a naso-gastric tube with an instantaneous drainage of 1000 ml of gastric content. A plain X-ray was performed to control the position of the naso-gastric tube which was adjusted to the gastric fundus. A naso-jejunal tube was placed by a guide-wire, passing the herniated and thereby intrathoracically positioned proximal duodenum (Fig. 2).

**Figure 3:**
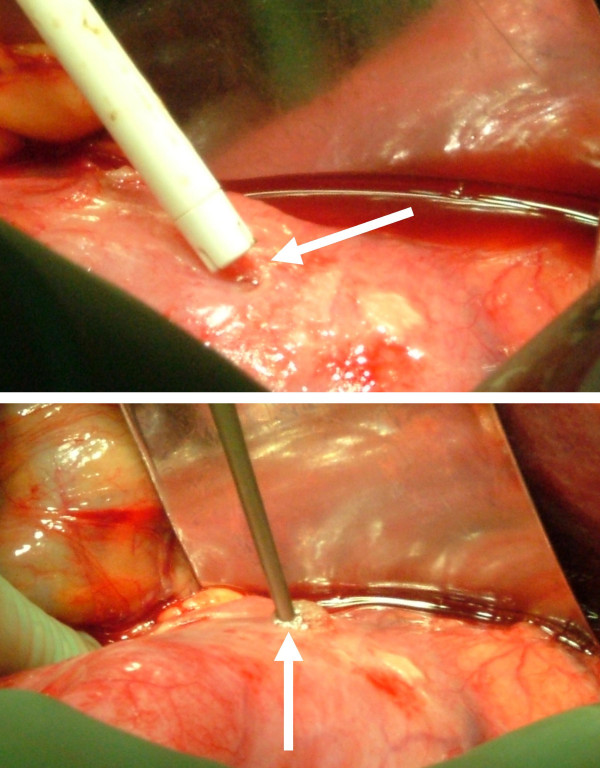
**Perforated peptic duodenal ulcer**. The ulcer was found to be a typically punched out peptic ulcer (arrows) with a diameter of 6 mm.

The patient was taken to surgery the following day. Through a mid-line incision, the cardia was located in normal anatomic position and laterally a severely distended gastric fundus was noted. On the right side of the esophagus, half of the transverse colon was found in a paraesophageal hernia, with a diameter of the crural defect of 5 cm. Behind the colon, the distal stomach and proximal duodenum was found to have been pulled up into the hernia. A phrenotomy was performed and the undamaged and viable colon was pulled out of the hernia. The gastro-duodenal transition was tapered to the colon. On the ventral part of the proximal duodenum there was a punched-out benign perforated peptic ulcer with a diameter of 6 mm (Fig. 3). By interrupted 3-0 Prolene^® ^sutures, the peptic ulcer perforation was closed and covered by a viable part of the omentum. A large portion of the omentum was found devitalized and was therefore resected. Due to bleeding, a splenectomy was performed. The devitalized hernial sac containing several septa was excised and an opening to the right pleura was made to allow drainage of the mediastinal cavity above the diaphragm by a chest tube. The right and left crura were mobilized and the diaphragmatic defect was closed by interrupted 2-0 Prolene^® ^sutures. To enforce the phrenoplasty and to minimize the risk of recurrence, a 360° Nissen fundoplication was performed. The patient received a jejunostomy tube for nutrition with the tip located 30 cm from the ligament of Treitz.

The patient was observed over-night in the ICU. To prevent infectious complications the patient received imipenem 500 mg × 3 i.v. for five days and as ulcer prophylaxis omeprazol 8 mg/h i.v. was given continuously. The chest-tube was removed on the fourth post-operative day. Eleven days after surgery the patient developed breathing problems with low saturation and tachycardia and a severe aspiration pneumonia was diagnosed and treated. The patient was discharged one month after surgery.

One month after discharge, the patient was followed-up at the out patient clinic and found to be in good shape and without nutritional problems. The jejunostomy tube was removed and the patient was put on life-long proton pump inhibition (T. Nexium^® ^40 mg × 1).

## Conclusion

Only a handful of papers have been published reporting perforation in a paraesophageal hernia and most of the reports cover gastric ulcer perforations with a postulated mechanical cause of the ulcer [8-13]. The mortality in the case of a perforated gastric ulcer in the context of paraesophageal hernia is very high [13]. To the best of our knowledge, only one former report exists concerning perforation of a peptic duodenal ulcer in combination with a paraesophageal hernia [14], and no report exits on a patient surviving this rare life threatening condition.

When not accidentally discovered, common presentations of paraesophageal hernias are postprandial discomfort, nausea, vomiting, hiccough, belching, dysphagia, reflux, chest gurgling, respiratory difficulties and cardiac type pain. Sub-acute or acute presentations of paraesophageal hernias, which may be life threatening, are gastrointestinal bleeding, gastric perforation, gastric or oesophageal obstruction, gastric volvulus or strangulation and thereby infarction of any herniated organ [6]. The risk of serious complications has, since many years, rendered in recommendations of early treatment of paraesophageal hernias by many influential authors [15,16]. In a recent study by Stylopoulos et al. [17], it is argued that this aggressive attitude should be modified since the pooled annual risk of developing acute symptoms from a paraesophageal hernia and thereby requiring emergency surgery is estimated to only about 1%. The lifetime risk of developing acute symptoms for a 65-year-old patient with a paraesophageal hernia is 18%, and the risk decreases exponentially with increasing age. Furthermore, in pooled data from the literature regarding emergency surgery in the situation of paraesophageal hernias, the mortality rate is calculated to 17%, which probably is an overestimation, since modern data base analysis has estimated the risk to 5.4% [17].

A perforated peptic ulcer requires, in most cases, immediate surgery and delay will increase the risk of death [18,19]. Patients with an episode of obstruction caused by a paraesophageal hernia also call for urgent surgery. However, if the stomach is decompressed by a naso-gastric tube, surgery may be postponed until a surgical team with experience of surgery from both above and below the diaphragm is at hand. According to our experience, gastric decompression may lower the risk of strangulation and reduce mechanical causes of gastric perforation. Furthermore, the risk of aspiration is decreased.

In the presented case, the hernial sac was contaminated with duodenal content and had to be removed. Due to the risk of an existing or iatrogenic small defect in to the pleural space, and as the common surgical dogma of not leaving contaminated areas undrained, we chose to drain the mediastinal cavity by a chest tube to avoid the formation of a concealed and undrained infection.

In conclusion, we hereby present a case with successful outcome, on the extremely rare life threatening combination of a paraesophageal hernia with a perforated peptic duodenal ulcer.

## Competing interests

The author(s) declare that they have no competing interests.

## Authors' contributions

ME: Management of the case and preparing the manuscript.

ER: Management of the case and critical appraisal and review of the manuscript.

JW: Interpretation of CT scan and X-rays

TZ: Management of the case and critical appraisal and review of the manuscript.

## Pre-publication history

The pre-publication history for this paper can be accessed here:
